# The *Leishmania amazonensis *TRF (TTAGGG repeat-binding factor) homologue binds and co-localizes with telomeres

**DOI:** 10.1186/1471-2180-10-136

**Published:** 2010-05-07

**Authors:** Marcelo S da Silva, Arina M Perez, Rita de Cássia V da Silveira, Camila E de Moraes, Jair L Siqueira-Neto, Lucio de H Freitas, Maria Isabel N Cano

**Affiliations:** 1 Telomeres Laboratory, Department of Genetics, Biosciences Institute, Universidade Estadual Paulista Júlio de Mesquita Filho, UNESP, Botucatu, SP, Brazil; 2 Center for Neglected Diseases Drug Discovery (CND3), Institut Pasteur Korea, Gyeonggi-do, South Korea

## Abstract

**Background:**

Telomeres are specialized structures at the end of chromosomes essential for maintaining genome stability and cell viability. The importance of telomeric proteins for telomere maintenance has increased our interest in the identification of homologues within the genus *Leishmania*. The mammalian TRF1 and TRF2 proteins, for example, bind double-stranded telomeres via a Myb-like DNA-binding domain and are involved with telomere length regulation and chromosome end protection. In addition, TRF2 can modulate the activity of several enzymes and influence the conformation of telomeric DNA. In this work, we identified and characterized a *Leishmania *protein (LaTRF) homologous to both mammalian TRF1 and TRF2.

**Results:**

LaTRF was cloned using a PCR-based strategy. ClustalW and bl2seq sequence analysis showed that LaTRF shared sequence identity with the *Trypanosoma brucei *TRF (TbTRF) protein and had the same degree of sequence similarities with the dimerization (TRFH) and the canonical DNA-binding Myb-like domains of both mammalian TRFs. LaTRF was predicted to be an 82.5 kDa protein, indicating that it is double the size of the trypanosome TRF homologues. Western blot and indirect immunofluorescence combined with fluorescence *in situ *hybridization showed that LaTRF, similarly to hTRF2, is a nuclear protein that also associates with parasite telomeres. Native and full length LaTRF and a mutant bearing the putative Myb-like domain expressed in bacteria bound double-stranded telomeric DNA *in vitro*. Chromatin immunoprecipitation showed that LaTRF interacted specifically with telomeres *in vivo*.

**Conclusion:**

The nuclear localization of LaTRF, its association and co-localization with parasite telomeres and its high identity with TbTRF protein, support the hypothesis that LaTRF is a *Leishmania *telomeric protein.

## Background

More than 20 *Leishmania *species are pathogenic to humans and cause leishmaniasis of differing severity. *Leishmania amazonensis *(Trypanosomatidae), the parasite studied in this work, is common in Brazil and causes a wide spectrum of clinical leishmaniasis [1]. The parasite can cause opportunistic infections in HIV/AIDS patients and co-infections have been reported in 34 countries. There are no adequate methods for controlling leishmaniasis and current available treatments are inefficient [2,3]. Consequently, most of the ongoing research for new drugs to combat the disease is based on post-genomic approaches [4].

Telomeres are specialized structures at the end of chromosomes and consist of stretches of repetitive DNA (5'-TTAGGG-3' in vertebrates and trypanosomatids) and associated proteins [5]. Telomeres are essential for maintaining genome stability and cell viability, with dysfunctional telomeres triggering a classic DNA-damage response that enables double-strand breaks and cell cycle arrest [6].

There are three classes of telomeric proteins, viz., proteins that bind specifically to single-stranded G-rich DNA, proteins that bind to double-stranded DNA and proteins that interact with telomeric factors. Other non-telomeric proteins, such as the DNA repair proteins Mre11 and Rad51, also play important roles at telomeres [7,8]. In mammals and yeast, telomeric proteins are organized in high order protein complexes known as shelterin or telosome that cap chromosome ends and protect them from fusion or degradation by DNA-repair processes [9,10,7]. These complexes, which are abundant at chromosome ends but do not accumulate elsewhere, are present at telomeres throughout the cell cycle and their action is limited to telomeres [7,8]. Shelterin/telosome proteins include members or functional homologues of the TRF (TTAGGG repeat-binding factor) or telobox protein family, such as TRF1 and TRF2 from mammals [11] and Tebp1 [12], Taz1 [13] and Tbf1 [14] from yeast. All of these proteins bind double-strand telomeres via a Myb-like DNA-binding domain, which is one of the features that characterize proteins that preferentially bind double-stranded telomeric DNA [15-17].

In humans, TRF1 may control the length of telomeric repeats through various mechanisms. For example, TRF1 can control telomerase access through its interaction with TIN2, PTOP/PIP1 and the single-stranded telomeric DNA-binding protein POT1. TRF1 may also regulates telomerase activity by interacting with PINX1, a natural telomerase inhibitor. In comparison, TRF2 is involved in many functions, including the assembly of the terminal t-loop, negative telomere length regulation and chromosome end protection [18,11,16]. The shelterin complex is anchored along the length of telomeres by both TRF2 and TRF1 [19], whereas in conjunction with POT1, TRF2 is thought to stimulate WRN and BLM helicases to dissociate unusual structures during telomeric replication [20]. TRF2 also interacts with enzymes that control G-tail formation, the nucleases XPF1-ERCC1, the MRE11-RAD50-NBS1 (MRN) complex, the RecQ helicase WRN and the 5' exonuclease Apollo [8]. Loss of TRF2 leads to NHEJ-mediated chromosome end-fusion and the accumulation of factors that form the so-called telomere dysfunction-induced foci (TIFs) [21,22]. Thus, TRF2 can modulate the activity of several enzymes and influence the conformation of telomeric DNA.

Only a few telomeric proteins that bind the double-stranded form of telomeric DNA have been described in *Leishmania *and in their trypanosome counterparts [17,23]. Homologues of human TRF have been found in the genomes of *T. brucei*, *T. cruzi *and *L. major *based on sequence similarities to the C-terminal Myb-like DNA binding domain. For example, the *T. brucei *TRF2 homologue known as TbTRF shares a similar telomere end-protection function with vertebrate TRF2 [24].

## Results and Discussion

### Characterization of the putative *L. amazonensis *TRF gene homologue

Using data mining via the OmniBLAST server we searched the whole *L. major *genome database http://www.ebi.ac.uk/parasites/leish.html for a putative sequence that shared similarities with the vertebrate TRF1 and TRF2 proteins. For this search, we used the most conserved part of both human proteins, the C-terminal fragment containing the Myb-like DNA binding domain. The search returned a single sequence (GenBank acc. no. XP_001682531.1) that encoded a hypothetical protein (GenBank acc. no. Q4QDR7, GeneDB_Lmajor **LmjF18.1250**), the C-terminus of which shared ~30% identity and 50-55% similarity with the vertebrate TRF Myb-like domain, according to the blast2 sequence analysis (Table 1).

**Figure 1:**
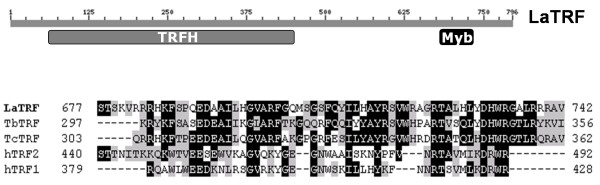
**LaTRF is a homologue of mammalian and *T. brucei *telomeric TRFs**. (top) Position of the TRFH and Myb domains in LaTRF, according to rpsblast and bl2seq sequence analysis with *T. brucei *TRF. (bottom) ClustalW multiple alignment of the Myb-like DNA binding domains of human (hTRF2 and hTRF1), *L. amazonensis *(LaTRF), *T. brucei *(TbTRF) and *T. cruzi *(TcTRF) TRFs.

Based on the *L. major *sequence, primers were designed for PCR amplification of the entire homologous sequence from *L. amazonensis *with genomic DNA as the template. PCR products of 2,931 bp were cloned into the vector pCR2.1 and both insert strands were sequenced (data not shown). The deduced polypeptide sequence of 796 amino acid residues contained a putative C-terminal Myb-like DNA binding domain between residues 684-733, according to psi-blast (Fig 1 - top). The *LaTRF *gene (GenBank acc. no. EF559263) shared high sequence identity and similarity to the putative *L. major *TRF, and to hypothetical *L. infantum *and *L. braziliensis *TRFs (Table 1). The sequence conservation between LaTRF and the trypanosome TbTRF and the putative TcTRF homologues decreased to 35-45% identity (Table 1), consistent with the known evolutionary relationships among these organisms. The *Leishmania *TRF homologues encode the largest TRF protein (~82.5 kDa) described so far. The fact that the *Leishmania *proteins showed much greater homology with each other than with other protozoan proteins and that they are the largest TRF described so far resembles the situation for *Leishmania *telomerase protein [25].

**Table 1:** Pairwise analysis of amino acid sequence alignments from TRF homologues based on bl2seq sequences (protein-protein BLAST)

	LaTRF (full length)		LaTRF^TRFHdomain^		LaTRF^Mybdomain^	
	
	%Identity	%Similarity	%Identity	%Similarity	%Identity	%Similarity
**LmTRF**	99	99	100	100	100	100

**LiTRF**	88	91	85	89	98	100

**LbTRF**	65	71	60	70	96	100

**TcTRF**	45	59	38	54	63	77

**TbTRF**	35	53	39	59	54	66

**hTRF1**	Not significant	Not significant	16	25	31	54

**hTRF2**	Not significant	Not significant	15	30	29	55

La, *L. amazonensis *(GenBank acc. no. EF559263); Lm, *L. major *(TrEMBL acc. no. **Q4QDR7**); Li, *L. infantum *(GenBank acc. no. XP_001464939.1); Lb, *L. brasiliensis *(GenBank acc. no. XP_001564056.1); Tc, *Trypanosoma cruzi *(GenBank acc. no. XP_819954.1); Tb, *Trypanosoma brucei *(GenBank acc. no. AY910010); h, human (hTRF1 GenBank Acc. no. P54274.2; hTRF2 GenBank acc. no. Q15554).

In addition, like TbTRF, LaTRF shared sequence similarities with the canonical Myb-like domain and with the TRFH dimerization domain of human TRF1 and TRF2 (Fig 1-bottom and Table 1), but no sequence similarities were found with any other telobox protein (data not shown). Together, these results indicate that although LaTRF shares high sequence similarity with TbTRF, probably because the two species are phylogenetically related [26], further studies are required to confer any functions to the *Leishmania *TRF homologue identified here.

### LaTRF is a nuclear protein that co-localizes with *L. amazonensis *telomeres

**Figure 2:**
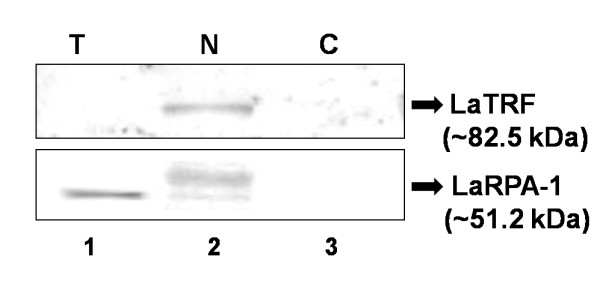
**Expression of LaTRF in *L. amazonensis *promastigotes extracts**. Western blot analyses of extracts from 10^7 ^promastigotes/lane, grown in mid-log phase, were probed with anti-LaTRF serum (top panel) and anti-LaRPA-1 serum [31] as the loading control (bottom panel). Lane 1 - total protein extract (T), lane 2 - nuclear extract (N), lane 3 - cytoplasmic extract (C).

In exponentially growing *L. amazonensis *promastigotes, LaTRF was detected only in nuclear protein extracts. A single ~82.5 kDa protein band was detected using anti-LaTRF serum (Fig 2 - top panel: lane 1). No protein was detected in cytoplasmic and total protein extracts (Fig 2 - top panel: lanes 2 and 3), indicating that LaTRF is a nuclear protein with very low intracellular abundance. As a control, Western blots were revealed with anti-LaRPA-1 serum, which recognizes a ~51.2 kDa telomeric protein band [23] (Fig 2 - bottom panel: lane 1) and also its phosphorylated forms (Fig 2 - bottom panel: lane 2; da Silveira & Cano, unpublished data).

**Figure 3:**
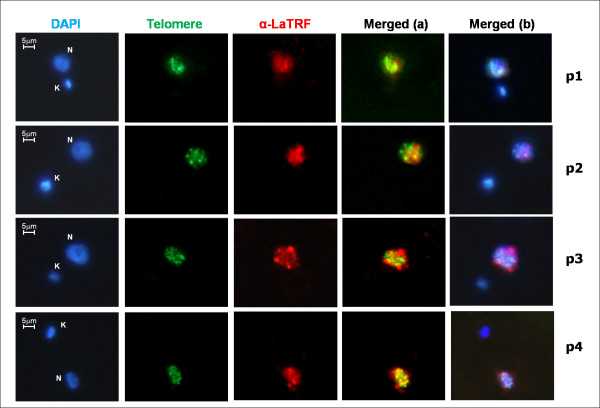
**LaTRF partially co-localizes with *L. amazonensis *telomeres**. LaTRF (red), using anti-LaTRF serum, was combined with FISH (green) using a PNA-telomere probe specific for TTAGGG repeats. DAPI (blue) was used to stain DNA in the nucleus (N) and in the kinetoplast (K). Images were organized in panels p1-p4 showing the co-localization patterns in merged (a): telomeres and LaTRF, and in merged (b): DAPI, telomeres and LaTRF. Merged images were done using NIS elements software (v. Br 2.30).

We also developed an immunofluorescence assay combined with FISH, using anti-LaTRF serum and a PNA-telomere probe specific for TTAGGG repeats. As shown in Fig 3 (panels p1-4, merged images a and b), LaTRF is a nuclear protein that partially co-localizes with parasites telomeres, since some of the LaTRF signal coincided with telomeric foci and some did not (Fig 3, panels p1-4). In most cells, LaTRF appears as a diffuse signal spread all over the nucleoplasm and only in some cases it forms large punctuated foci, which seems to co-localize with the telomeric DNA (yellow dots in Fig 3, panels p2 and p4). Similarly, in humans, the hTRF2 protein also appears in the form of punctuate foci that does not completely associate with telomeres [18], which is in agreement with other cellular functions played by this protein [27,28]. In contrast, the *T. brucei *TRF protein (TbTRF) appears to co-localize with most telomeres at all stages of the cell cycle in both bloodstream and procyclic forms [24]. Whether LaTRF also has other cellular roles or if its association with telomeres occurs in a cell cycle dependent manner is not clear at this stage.

### LaTRF interacts in vitro and in vivo with *L. amazonensis *telomeres using a Myb-like DNA binding domain

**Figure 4:**
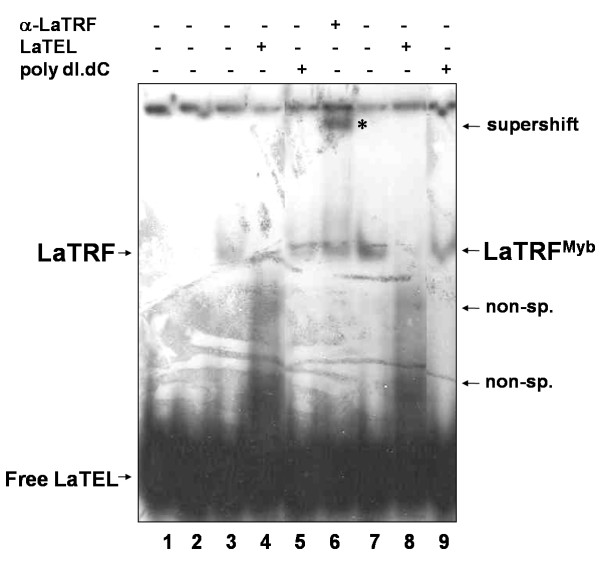
**Recombinant LaTRF and the mutant bearing the C-terminal Myb domain bind *in vitro *double-stranded telomeric DNA**. Electrophoretic mobility shift assays (EMSA) were done using radiolabeled double-stranded telomeric DNA (LaTEL) as probe. Protein:DNA complexes were separated in a 4% PAGE in 1X TBE. EMSA was done with *E. coli *BL21 protein extract (lane 2), recombinant full length LaTRF (lanes 3-6) and a mutant bearing the C-terminal Myb domain (lanes 7-9). A supershift assay was done with anti-LaTRF serum (lane 6). Assays were also done in the presence of 20 fold excess of non-labeled LaTEL as specific competitor (lanes 4 and 8) or 100 fold excess of double-stranded non-specific poly [dI-dC] [dI-dC] DNA (lanes 5 and 9). In lane 1, no protein was added to the binding reaction. The original gel image and its content are shown as additional file 1: Figure S1.

**Figure 5:**
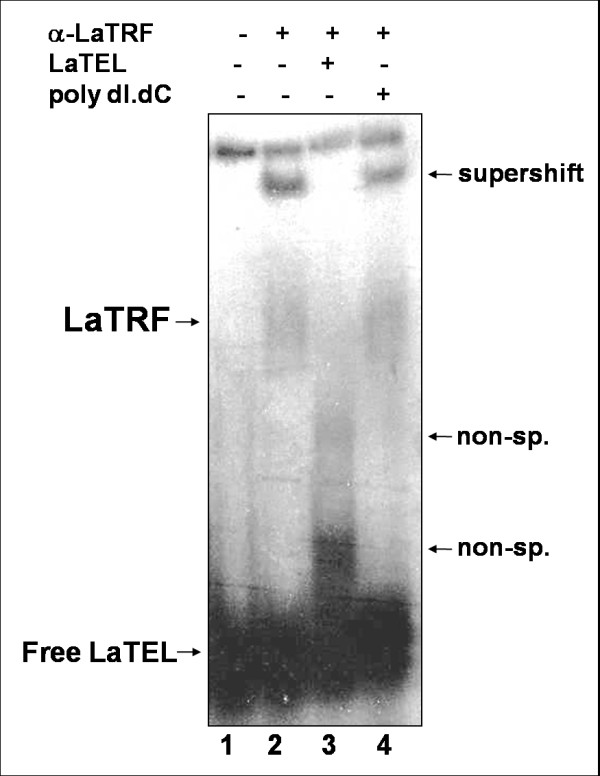
**Supershift and competition assays confirm that recombinant full length LaTRF bind *in vitro *double-stranded telomeric DNA**. Electrophoretic mobility shift assays (EMSA) were done using radiolabeled double-stranded telomeric DNA (LaTEL) as probe. Protein:DNA complexes were separated in a 4% PAGE in 1X TBE. EMSA was done with recombinant full length LaTRF and anti-LaTRF serum in the absence (lane 2) and in the presence of 20 fold excess of non-labeled LaTEL as specific competitor (lane 3) or 100 fold excess of double-stranded non-specific DNA (poly [dI-dC] [dI-dC]) as non specific competitor (lane 4). In lane 1 reaction was done in the presence of LaTEL only.

**Figure 6:**
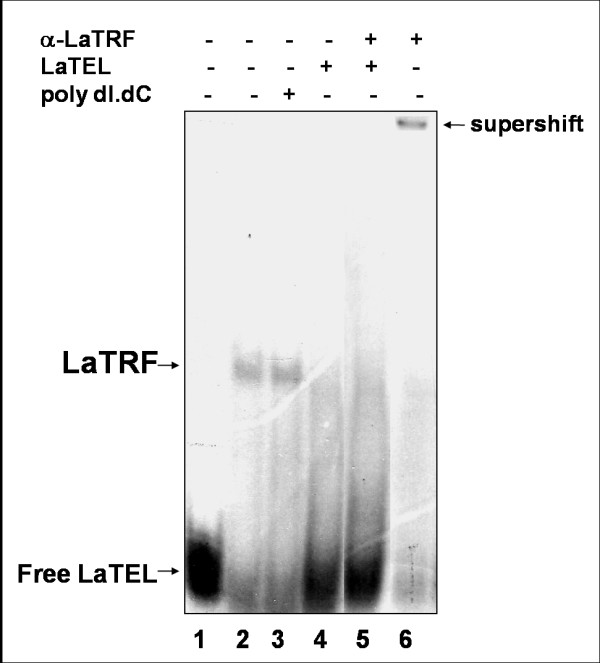
**Nuclear extracts obtained from *L. amazonensis *promastigotes contain LaTRF bind activity**. Electrophoretic mobility shift assays (EMSA) were done using radiolabeled double-stranded telomeric DNA (LaTEL) as probe. Protein:DNA complexes were separated in a 4% PAGE in 1X TBE. In lanes 2-6, EMSA was done with nuclear extracts obtained from *L. amazonensis *promastigotes. In lane 2, the reaction was done in the absence of competitors. In lanes 3 and 4, binding reactions were done respectively, in presence of 100 fold excess of double-stranded non-specific DNA (poly [dI-dC] [dI-dC]) and 20 fold excess of non-labeled LaTEL. In lane 5, a supershift assay was done with anti-LaTRF serum and in the presence of 20 fold excess of non-labeled LaTEL and in lane 6, the supershift assay shown in lane 5 was done in the absence of competitors.

EMSA assays were done with renatured protein extracts containing full length LaTRF, the Myb-like DNA binding domain (LaTRF^Myb^) (Figs 4 and 5, see additional file 1) and with *L. amazonensis *nuclear extracts (Fig 6), to investigate whether LaTRF, like its vertebrate and trypanosome counterparts [18,24], was able to bind double-stranded telomeric DNA *in vitro*.

The full-length recombinant protein and its deletion mutant were expressed in very low amounts and in non-soluble form in the *E. coli *system (data not shown) making their purification by conventional chromatography very difficult. Therefore, protein expression was checked by Western blot using anti-LaTRF serum and anti-His tag monoclonal antibody (data not shown).

As shown in Fig 4, recombinant full length LaTRF and the mutant bearing only the C-terminal Myb-domain were able to bind specifically the double-stranded telomeric DNA (LaTEL). Competition assays showed that the complexes formed by both recombinant proteins were completely abolished in the presence of excess unlabeled LaTEL and that there was no competition for binding when excess of non-specific poly [dI-dC] [dI-dC] double-stranded DNA was used (Fig 4, lanes 4, 5, 8 and 9). Supershift assay with anti-LaTRF serum, which recognizes a N-terminal epitope in the protein, confirmed that full length LaTRF forms a robust complex with labeled LaTEL (Fig 4, lane 6), possibly because the binding of anti-LaTRF stabilized the LaTRF-LaTEL complex, blocking the action of other non-specific binding activity in the extract. When competitors were added to the supershift reactions with anti-LaTRF serum, the binding specificity of recombinant LaTRF for LaTEL was confirmed (Fig 5, lanes 2-4). The complex was almost totally abolished in the presence of excess unlabeled LaTEL (Fig 5, lane 3) and no competition was detected in the presence of non-specific DNA (Fig 5, lane 4). The results presented above suggest that recombinant LaTRF binds LaTEL potentially via the putative Myb-like DNA binding domain indicating a role for the C-terminal region of LaTRF in mediating sequence-specific binding to telomeric DNA.

Nuclear extracts were obtained from log phase *L. amazonensis *promastigotes in order to check if native LaTRF was also able to bind double-stranded telomeric DNA (LaTEL) *in vitro*, (Fig 6). The results showed the presence of LaTRF activity in these extracts, as part of the complex formed with the nuclear proteins and LaTEL (Fig 6, lane 2) was supershifted by the anti-LaTRF serum (Fig 6, lane 6). In addition, competition assays showed that this complex was unaffected by excess of poly [dI-dC] [dI-dC] (Fig 6, lane 3), used as the non-specific competitor, but it was almost completely abolished in the presence of excess unlabeled LaTEL (Fig 6, lane 4). Supershift experiments using anti-LaTRF serum were done in the presence of competitor to confirm that LaTRF was actually involved in the formation of the retarded band (Fig 6, lane 5). Note that the retarded shifted band disappeared due to the competition by non-labeled LaTEL. Thus, these results indicate that LaTRF is in part responsible for the binding activity shown in these extracts and is probably a component of the *Leishmania *telomeric complex.

**Figure 7:**
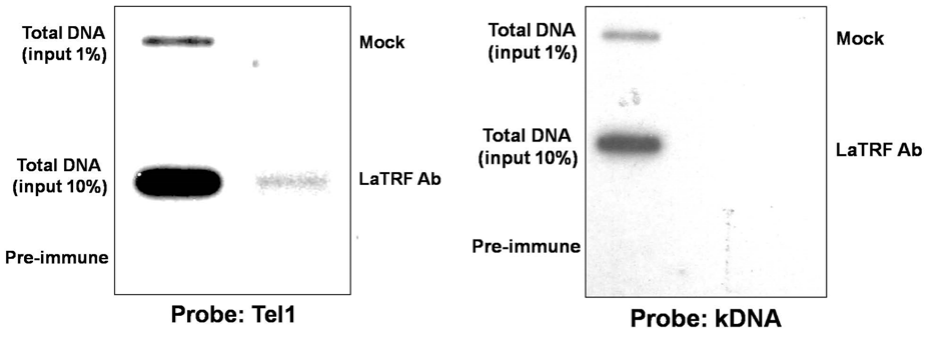
**LaTRF interacts with telomeric DNA *in vivo***. Chromatin immunoprecipitation (CHIP) of mid-log phase promastigotes cells using anti-LaTRF. Control experiments were done with chromatin immunoprecipitated in the presence of pre-immune serum and without serum (mock). Total DNA (input) corresponds to 10% and 1% of the amount of DNA in 10^8 ^cells cross-linked with the chromatin. Slot-blots were hybridized with 5' end-labeled Tel1 probe (left) and re-hybridized with the kDNA probe (right).

Chromatin immunoprecipitation experiments also suggested that LaTRF is a telomeric protein. The anti-LaTRF serum immunoprecipitated *L. amazonensis *telomeric DNA (Tel1) *in vivo *(Fig 7 - left) but did not immunoprecipitate the GT-rich kinetoplast DNA (kDNA) (Fig 7 - right). The kDNA control represented by the UMS (universal mini-circle sequence) albeit GT-rich, is very representative of the general base composition of *Leishmania *genomic DNA. In addition, it is a good control, since we were able to show that it was co-immunoprecipitated by two other *Leishmania *telomeric protein [17,23]. In a previous study, we described LaTBP1, a protein that specifically binds telomeric and GT-rich DNA in *Leishmania*. LaTBP1 has a centrally positioned Myb-like DNA binding domain and is most likely a non-telobox protein that is apparently related to the multifunctional yeast RAP1 telomeric protein and TFIIIB B" transcription factor [17]. Together with the putative LaTRF described here, these are the only descriptions of proteins bearing a Myb-like DNA binding domain that interact with double-stranded telomeric DNA in *Leishmania*.

As mentioned here and elsewhere [26], the huge evolutionary distance between this protozoan and higher eukaryotes presents a barrier when searching for protein homologues in the genomes of these parasites. For example, no TRF1 homologues were found in trypanosomatid genomes but the expression of hTRF1 in procyclic forms of *T. brucei *caused telomere shortening and cell cycle arrest, probably by displacing an unknown endogenous telomeric factor [29]. RNAi knockdown of TbTRF arrested bloodstream cells in G2/M and most of the procyclic cells in the S phase, and caused shortening of G-rich single-stranded telomeric DNA. These findings suggest that TbTRF is probably the unknown endogenous telomeric factor, which resembles the function of mammalian TRF2 at parasite telomeres [24]. The functions of LaTRF at *Leishmania *telomeres remain to be determined.

## Conclusions

In this report we describe the characterization of the *Leishmania *TRF homologue and show that it is the largest TRF protein homologue described so far. This protein contains a canonical C-terminal Myb-like DNA binding domain as well as a putative and less conserved TRFH dimerization domain [30]. In addition, LaTRF is expressed exclusively in the nucleus and like its vertebrate and trypanosome counterparts, binds to parasite telomeres *in vitro *and *in vivo*. It can also co-localize with parasite telomeres, despite being spread all over the nucleoplasm in most cells, suggesting that LaTRF may play additional cellular roles beyond its possible telomeric function.

## Methods

### Parasite cultures

*L. amazonensis *promastigotes (MHOM/BR/73/M2269) were grown in M199 medium (Cultilab) supplemented with 10% fetal calf serum (Cultilab), 25 mM HEPES and 1 × antibiotic/antimycotic solution (Cultilab) at 28°C.

### Isolation of *L. amazonensis *genomic DNA and cloning of the *LaTRF *gene

Total genomic DNA of *L. amazonensis *was prepared as previously described [31]. LaTRF was cloned using a PCR-based strategy. Primers were designed based on the putative sequence LM16.2.Contig67 from *L. major *(GeneDB_Lmajor **LmjF18.1250**) for amplification of the complete LaTRF open reading frame (ORF) (See additional file 2: Table S1). The PCR product spanning the entire *L. amazonensis *TRF ORF (2,391 bp) was obtained by using the primers F1 and R1 and 1U of Platinum *Taq *(Invitrogen) followed by cloning into the pCR^® ^2.1 cloning vector (Invitrogen). The PCR product was sequenced using specific primers and primers from the vector (See additional file 2: Table S1). The primers F1 and R1 contained restriction sites for *Nde*I and *Xho*I (See additional file 2: Table S1) to allow further cloning of the gene in-frame with a N-terminal 6× His-tag into plasmid pET-28a+ (Novagen).

Amino acid sequence alignments were done with blastp, bl2seq, cds http://blast.ncbi.nlm.nih.gov/Blast.cgi and ClustalW http://www.ebi.ac.uk/clustalw/ using default parameters. The sequences used for these analyses were: hTRF2 (GenBank acc. no. Q15554), hTRF1 (GenBank acc. no. P54274.2), TbTRF (GenBank acc. no. AY910010), putative LmTRF (TrEMBL acc. no. **Q4QDR7, **GeneDB_Lmajor **LmjF18.1250**), TcTRF (GenBank acc. no. XP_819954.1), LiTRF (GenBank acc. no. XP_001464939.1) and LbTRF (GenBank acc. no. XP_001564056.1). The *L. amazonensis LaTRF *gene sequence was submitted to GenBank and is available under the accession number EF559263.

### Construction of an LaTRF deletion mutant (*LaTRF*^*Myb*^)

To verify the existence of a Myb-like DNA-binding domain at the C-terminus of the protein, a deletion mutant was constructed. The primers F3 and R1 (See additional file 2: Table S1) were used to amplify the deletion mutant *LaTRF*^*Myb *^from genomic DNA, which contained the putative C-terminal Myb-like DNA binding domain. This mutant has approximately 665 bp that span nt 1726-2391. As with full length *LaTRF*, the *LaTRF*^*Myb *^mutant was cloned into the pCR^® ^2.1 cloning vector (Invitrogen), sequenced and subcloned into a pET28a+ expression vector.

### Expression of LaTRF and the deletion mutant LaTRF^Myb ^proteins in *E. coli*

Full length *LaTRF *and the deletion mutant *LaTRF*^*Myb*^cloned into a pET 28a+ vector, were transformed in *E. coli *strain BL21 DE3 RP codon plus cells for expression in the presence of 1 mM IPTG. Both proteins were expressed in low amounts and in non-soluble form, preventing them from being purified by affinity chromatography based on the 6× His-tag. To overcome this problem, the non-soluble bacterial pellets containing both proteins were solubilized in 7 M urea, sonicated in the presence of 10 U of DNAse I (Sigma) and renatured by dialysis in 50 mM glycine, pH 8.0. The presence of each protein in the extracts was checked by electrophoresis in 10% SDS-PAGE followed by Western blot probed with anti-LaTRF serum and with an anti-His tag monoclonal antibody (Novagen).

### Preparation of *L. amazonensis *total and nuclear extracts

Promastigotes in mid-exponential growth were used to obtain both extracts. Nuclear and cytoplasmic extracts were prepared with a Nuclear Extract Kit (Active Motif) adapted for *L. amazonensis *promastigotes in the presence of phosphatase and protease inhibitors.

Total protein extracts were obtained using RIPA buffer (150 mM Tris-HCl pH 7.5, 150 mM NaCl, 1% Triton X-100 and 0.1% SDS) in the presence of 10 U of DNase I and 1X protease inhibitor cocktail (Calbiochem) and incubated for 15 min at 4°C. Cell lysates were homogenized by vortexing at maximum speed (5 bursts of 10 s each). Extracts were cleared by centrifugation at 9,300 ×g for 8 min at 4°C, to separate the total protein (supernatant) from the cellular debris (pellet).

Both extracts were stored at -80°C and their protein concentrations were measured by the Bradford dye-binding assay, using bovine serum albumin as standard.

### Western blot analysis

Different protein extracts obtained from 10^7 ^parasites were separated by SDS-PAGE on 10% polyacrylamide gels and transferred to nitrocellulose membranes (BIO-RAD) in Tris-glycine-methanol at 16°C. The membranes were probed with rabbit anti-TRF2 serum raised against the synthetic peptide Nt-APAVTTRKRPRSSDSP-Ct (Sigma). The extracts were also probed with anti-LaRPA-1 serum as a control [23,32]. In both cases, immunoreactive bands were revealed by using an Amplified Alkaline Phosphatase Immun-Blot Assay Kit, according to the manufacturer's instructions (BIO-RAD).

### Indirect immunofluorescence combined with Telomere PNA FISH (fluorescence in situ hybridization)

This assay was performed using previously described protocols [32,33] with minor modifications. For IF, 10^6 ^promastigotes cells were washed in 1X PBS and fixed in 1% formaldehyde for 5 min at 4°C. After permeabilization with 0.1% Triton X-100 (in 1X PBS) for 10 min at room temperature, cells were incubated with 0.1 M Glycine (in 1X PBS) and attached to glass coverslips coated with 0.1% poly-L-Lysine (Sigma). Anti-LaTRF serum was used to detect LaTRF with Alexa Fluor 555-labeled goat anti-rabbit IgG (Invitrogen) as the secondary antibody followed by telomere detection using a Telomere PNA FISH Kit/FITC (DakoCytomation). VECTASHIELD^® ^Mounting Medium with DAPI (Vector Labs) was used as the anti-fade mounting solution and to stain nuclear and kinetoplast DNA. The images were analyzed with a Nikon 80i fluorescence microscope and captured with a digital camera (Nikon). When necessary, images were superimposed using NIS elements software (v. Br 2.30).

### EMSA (electrophoretic mobility shift assay)

All of the conditions for binding reactions and EMSA, including binding temperature, protein concentrations in the extracts and the double-stranded DNA probe (LaTEL), were standardized in preliminary experiments. LaTEL was constructed by using the γ [^32^P]ATP 5'-end-labeled oligonucleotides ssTel78G and ssTel78C, as described by Lira *et al*. [17]. Assays were done by mixing 10 μg of renatured bacterial extracts containing full length LaTRF or LaTRF^*Myb *^with approximately 2 pmol of labeled probe (LaTEL) in 30 μl of EMSA buffer (20 mM HEPES, 2.5 mM MgCl_2, _0.1 mM EDTA, 0.1 M KCl, 10% glycerol, 0.5 mM DTT, pH 8.0) containing 10 ng of poly [dI-dC] [dI-dC] and 10 ng of poly [dA-dT] [dA-dT]. Total protein extracts of non-transformed *E. coli *were used as controls. The reactions were incubated for 30 min at room temperature and loaded onto a non-denaturing 4% polyacrylamide gel (acrylamide:bis-acrylamide, 19:1, w/w) in 1X TBE. After electrophoresis, the gels were exposed to X-ray film.

Binding reactions were also done with crude nuclear extracts obtained from 10^8 ^parasites (~2.3 μg of total proteins) and γ [^32^P]ATP labeled LaTEL (2 pmol) in EMSA buffer containing a mixture of 10 ng of poly [dI-dC] [dI-dC] and 10 ng of poly [dA-dT] [dA-dT].

Competition assays to test the binding specificity of proteins in both recombinant and nuclear extracts, were done using 20 fold excess of unlabeled LaTEL (in relation to the labeled probe) as the specific competitor and a 100 fold excess (in relation to the labeled probe) of unlabeled double-stranded DNA poly [dI-dC] [dI-dC] as the non-specific competitor. Supershift assays were done using full-length recombinant LaTRF (10 μg) or native nuclear extracts from 10^8 ^parasites in the presence of ~30 μg of anti-LaTRF serum in EMSA buffer containing labeled LaTEL as probe and both poly [dI-dC] [dI-dC] and poly [dA-dT] [dA-dT] as above described. These assays were also performed in the presence of 20 fold excess of non-labeled LaTEL and 100 fold excess of poly [dI-dC] [dI-dC] as described above.

### Chromatin immunoprecipitation

Formaldehyde cross-linked chromatin was obtained from promastigote forms of *L. amazonensis *parasites (0.8 × 10^8 ^cells/experiment) as described by Lira et al. [17]. Chromatin was immunoprecipitated with anti-LaTRF serum and DNA was extracted after cross-link reversal. DNA samples were slot-blotted and hybridized with Tel1 and kDNA probes by using a previously established protocol. Aliquots of 1% and 10% of total DNA used in each experiment (input) were tested separately. Control assays included immunoprecipitation of chromatin with pre-immuneserum (pre-immune) or without serum (mock). The probes used were 5'-end labeled with γATP [^32^P]: Tel1 (5'TTAGGG-3')_3 _and kDNA (5'-TTTCGGCTCGGGCGGTGAAAACTGGGGGTTGGTGTAAAAT-3'), according to Lira *et al*. [17].

## Authors' contributions

MSS performed molecular cloning techniques, designed the deletion mutant, produced recombinant proteins, participated in the sequence alignment analysis, standardized the IF/FISH assays and has been involved in drafting the manuscript. AMP participated in the production of recombinant proteins, performed *in vitro *binding assays and has also been involved in drafting the manuscript. RCVS and CEM obtained native protein extracts and performed Western blots and chromatin immunoprecipitation assays. JLSN helped MSS with the cloning strategies, IF/FISH experiments and designed the peptide used to generate anti-LaTRF serum. LHFJ collaborated in outlining some experimental strategies and has been involved in the manuscript revision contributing with important intellectual content. MINC coordinated and designed most of the experiments as well as the strategies used in the manuscript, has mentored MSS, AMP, RCVS and CEM, who have also contributed during discussions of the results. MINC critically read and reviewed the manuscript for its publication. All authors read and approved the final manuscript.

## Supplementary Material

Additional file 2 **Table S1 Primers used for PCR amplification and sequencing of the putative L. amazonensis TRF gene and the deletion mutant LaTRFMyb**. Table containing a list of the primers used for PCR and sequencing assays Click here for file

Additional file 1 **Figure S1. Original and unmanipulated gel image shown in figure 4.** EMSA done with radiolabeled double-stranded telomeric DNA (LaTEL) as probe. Protein:DNA complexes were separated in a 4% PAGE in 1X TBE. In lane 1, no protein was added to the binding reaction. In lane 2, EMSA was done with E. coli BL21 protein extract. In lane 3, EMSA was done with recombinant full length LaTRF. In lane 4, EMSA was done with recombinant full length LaTRF in the presence of 20 fold excess of non-labeled LaTEL as specific competitor. In lane 5, no protein was added to the binding reaction (as in lane 1). In lane 6, EMSA was done with recombinant full length LaTRF in the presence of 100 fold excess of double-stranded non-specific poly [dI-dC] [dI-dC] DNA. In lane 7, EMSA was done with recombinant full length LaTRF in the presence of anti-LaTRF serum (supershift assay). Please check the supershifted complex at the top of the lane. In lane 8, EMSA was done with the mutant recombinant protein bearing the C-terminal Myb domain. In lane 9, EMSA was done with the mutant recombinant protein bearing the C-terminal Myb domain in the presence of 20 fold excess of non-labeled LaTEL. In lane 10, the same experiment shown in lane 9. In lane 11, EMSA was done with the mutant recombinant protein bearing the C-terminal Myb domain in the presence of 100 fold excess of double-stranded non-specific poly [dI-dC] [dI-dC] DNA. In lane 12, the same supershift assay shown in lane 7. Click here for file
